# Progress in Dermatology

**Published:** 1904-03-12

**Authors:** 


### Progress in Dermatology.

Savill[1] has met with seven different kinds of hysterical skin symptoms: (1) Attacks of generalised pallor of the surface; (2) attacks of flushing; (3) fugitive localised patches of congestion suddenly appearing and disappearing; (4) dermatographia; (5) acroparæsthesia and erythromelalgia; (6) urticarial, erythematous, and hæmorrhagic exudations; and (7) localised ischæmia. The last four he views as a vasomotor neurosis plus toxæmia. Flushing he regards as the most important of the stigmata of the hysterical diathesis. "A sudden oscillatory dilatation or constriction of a visceral vascular area (splanchnic or thoracic), initiated in most cases by an emotional condition of the brain, and this is attended by a corresponding constriction or dilatation of the surface vessels. Acroparæsthesia and erythromelalgia are most frequently met with in hysterical subjects and in the female sex. Attacks of deadly-white pallor of the fingers are more common in hysterical subjects. Exudative skin conditions are far more frequently met with in hysterical subjects. Evidences of hysterical diathesis can be found in 85 to 90 per cent. of his hospital cases of urticaria factitia, all kinds of erythema and circumscribed œdema. There are at least three ways in which these exudative skin conditions can be produced: (1) By chemical changes in the blood; (2) by nervous influences (nervo-vascular and emotional); (3) by a combination of these two. The last named comprises most of the exudative skin conditions. In urticaria (serous exudation) not all cases are due to toxæmia, some being directly attributable to nervous influence. The same may be said of erythema multiforme, erythema nodosum and pernio, in all of which there is a sero-sanguineous exudation, as also in urticaria pigmentosa. Certain cases of hæmorrhagic or purpuric effusions have less connection with hysteria than the preceding, but the connection does exist. In a case of localised ischæmia he tried calcium chloride, 15 grs. thrice daily with ammonium bromide, with good results. Summing up he says, "Among the co-operative causes sometimes in operation may be mentioned toxins of gastro-intestinal origin, articles of diet, traumatism, toxins of insects, and other causes. But without the vasomotor instability, innate—*i.e.* hysterical—or acquired, these cannot act." Finally he draws attention to the connection between the skin and the central nervous system from a developmental standpoint; and instances syphilis as affording a striking illustration of their pathological resemblance. This disease is specially apt to affect both the skin and the central nervous system in preference to, though not to the exclusion of, the other parts of the body. "Undoubtedly," he says, "the places in which to study syphilis are in a skin and in a nerve clinic—and these, if I mistake not, are also the best places in which to study hysteria."

Osler,[2] in adding a further series of cases of the "visceral manifestations of the erythema group of skin diseases, considers that cerebral affections, occurring during erythematous attacks, may be explained by attributing them to vascular changes in the brain, the counterpart of those occurring in the skin. He quotes as an analogous condition that met with in Raynaud's disease, where repeated attacks of monoplegia, aphasia, and hemiplegia have been known to alternate or be simultaneous with local syncope or asphyxia of the fingers and toes. This paper instances just such cases as those of which Crawford treats (v. infra) under the title of Erythema exsudativum multiforme. As to treatment, he states that for very chronic cases with recurring colic, though these may resist all measures, a course of grey powder may be helpful. With angioneurotic œdema nitro-glycerine in full doses may be tried. In one case camphor did good. The chief danger lies in the nephritis, against which he urges protracted rest in bed and a milk diet as the best means at our disposal.

Raymond Crawford[3] records three cases of erythema exsudativum multiforme which showed symptoms of purpura symmetrically arranged, articular and periarticular effusions, attacks of abdominal pain, hæmorrhages from mucous membranes and kidneys, etc., and irregular febrile temperature. He criticises the term "Henoch's purpura" as misleading, in that the symptoms which, grouped together, form the condition known by this name comprise "merely one manifestation of a morbid state which exhibits protean changes of form." There is evidence of a toxæmia, but cultures from the blood of his cases were all negative. This toxæmia, like that of scarlet fever, spends itself with varying force on the skin, the kidneys, the heart, and the joints, and which in its cutaneous features presents a marked symmetry.

The skin affections of certain cases of trypanosomiasis are given by Galloway.[4] The rash is most pronounced at the invasion, but is of long duration. There is a slight general œdema of the skin, which is partly subcutaneous. On this skin is seen a polycyclical erythema, the diameters of the segments of the circles being from $\frac{1}{2}$ to many inches. Slight pigmentation is left. The eruption is general, but appears to affect the back of the trunk and the face in a marked degree. The eruption presents an appearance quite unusual in other erythematous disease. There is a peculiar irritable papulo-vesicular eruption in sleeping sickness, which disease, being due to the presence of the trypanosoma in the cerebro-spinal fluid, adds additional interest to the above-mentioned erythema. In speaking of lupus erythematosus, he adds his weight to the suggestion that the disease is a peculiar form of exudative erythema characterised by special lesions of the nature of chronic œdema and by the appearance of cells in the exudation which have a longer life-period and different functions than ordinary leucocytes (plasma cells). He regards toxæmia as the cause, arising from various sources, as in erythema multiforme. There is, however, a strong indication of a second factor besides the vasomotor disturbance produced by the toxin—viz. the tendency for easily produced paralysis of the vasomotor mechanism.

Hollander[5] has treated successfully some of the severest and most unfavourable cases of lupus erythematosus by means of large doses of quinine internally ($\frac{1}{2}$ gramme of sulphate or hydrochlorate thrice daily), and 5 to 10 minutes after each dose the affected parts are painted with iodine. This is repeated for five to six days. The crusts produced are allowed to separate, and the result is a scar-like atrophy or a complete return to normal. In the majority of cases about 60 grammes of quinine were necessary to complete the cure.

1 Lancet, Jan. 30, 1904. 2 Amer. Jour. Med. Sci., Jan., 1904. 3 Lancet, Oct. 24, 1903. 4 Brit. Med. Jour., July 18, 1903. 5 Amer. Jour. Med. Sci., May, 1903.

*(To be continued.)*

